# Automated Detection of Iris Furrows and their Influence on Dynamic Iris Volume Change

**DOI:** 10.1038/s41598-017-18039-w

**Published:** 2017-12-20

**Authors:** Jacqueline Chua, Sri Gowtham Thakku, Tan Hung Pham, Ryan Lee, Tin A. Tun, Monisha E. Nongpiur, Marcus Chiang Lee Tan, Tien Yin Wong, Joanne Hui Min Quah, Tin Aung, Michael J. A. Girard, Ching-Yu Cheng

**Affiliations:** 1 Singapore Eye Research Institute, Singapore National Eye Centre, Singapore, Singapore; 2 Ophthalmology & Visual Sciences Academic Clinical Program (Eye ACP), Duke-NUS Medical School, Singapore, Singapore; 3 Department of Biomedical Engineering, National University of Singapore, Singapore, Singapore; 4 Department of Ophthalmology, Yong Loo Lin School of Medicine, National University of Singapore and National University Health System, Singapore, Singapore; 5 SingHealth Polyclinics, Outram, Singapore

## Abstract

We introduced a new method for detecting iris surface furrows and identify its associations with dynamic changes in iris volume in healthy eyes. Swept-source optical coherence tomography was performed on 65 subjects with open angle under light and dark conditions. Iris boundaries were identified and a reconstruction of the anterior iris surface was obtained. Furrows were detected by identifying locally deep (minima) points on the iris surface and reported as furrow length in millimetres. Iris volume was quantified. Associations between furrow length and dynamic changes in iris volume were assessed using linear regression model. With pupil dilation, furrow length increased (15.84 mm) whereas iris volume decreased (−1.19 ± 0.66 mm^3^). Longer furrow length was associated with larger static iris volume, as well as smaller loss of iris volume with pupil dilation (β = −0.10, representing 0.1 mm^3^ less loss in iris volume per 10 mm increase in iris furrow length; *P* = 0.002, adjusted for age, gender and changes in pupil size). Our iris furrow length measurements are robust and intuitive. Eyes with longer furrows have larger iris volume and lose less volume during physiological pupil dilation. These findings highlight the potential for iris surface features as indicators of iris morphological behavior.

## Introduction

Primary angle closure glaucoma (PACG) is a major cause of blindness worldwide^1,2^ and is particularly prevalent among people of Chinese and East Asian ethnicity^3–6^. While several risk factors for PACG have been identified^7–9^, including shorter axial length, narrower and shallower anterior chamber, recent studies have also shown that the dynamic changes in iris volume may play an important role in angle closure^10–15^. In particular, closed angles may be associated with a smaller change in iris volume during pupil dilation^12–14^.

**Figure 1:**
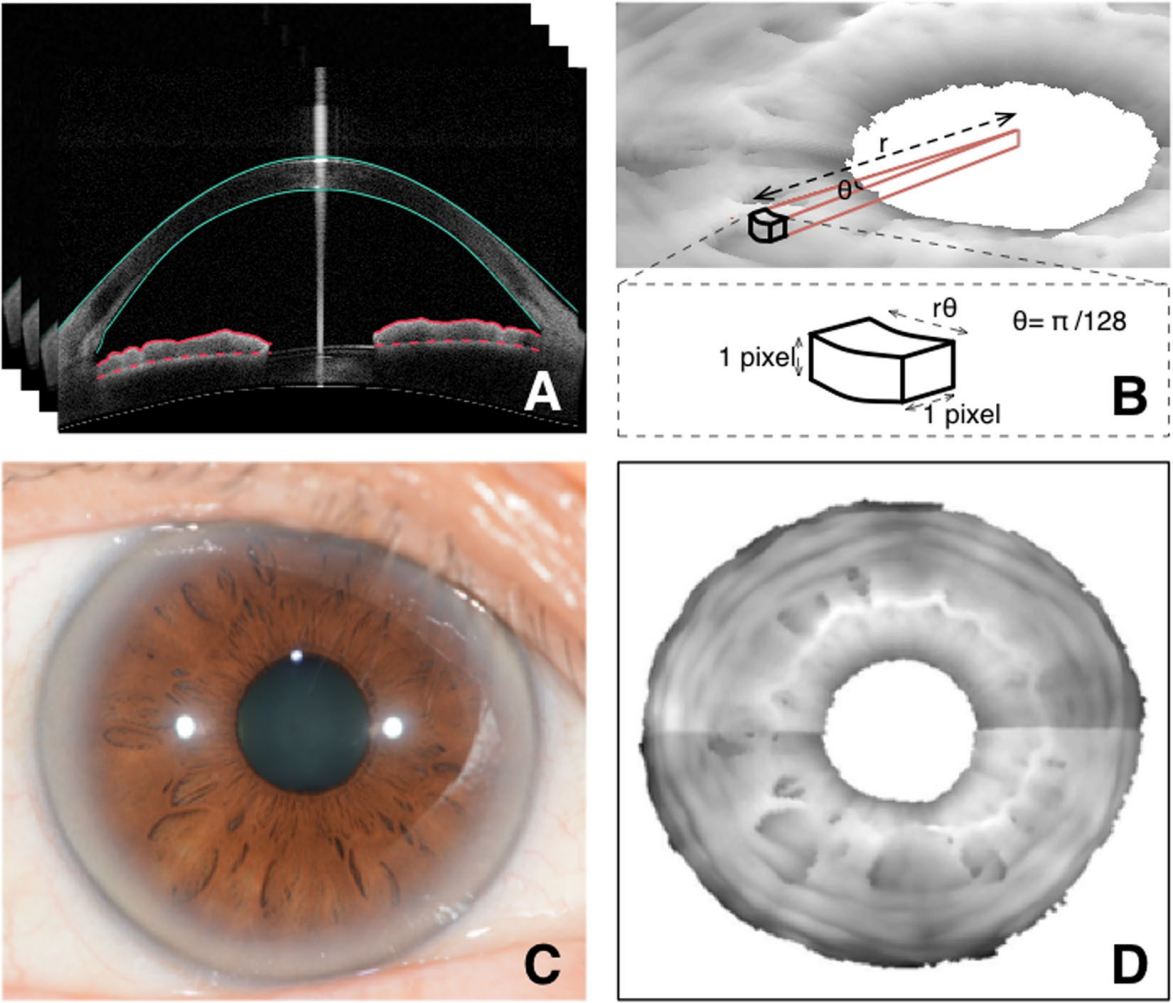
(**A**) Anterior (red solid) and posterior (red dashed) iris boundaries detected on radial SS-OCT scans of the anterior segment; (**B**) A three dimensional reconstruction of the anterior iris surface is obtained based on the iris boundary detection on 128 radial SS-OCT scans of each eye also showing the dimensions of one voxel; the circumferential distance of the voxel is proportional to ‘r’, its distance from the pupil center; (**C**) Digital photograph of the scan eye shows arcus senilis in the peripheral iris, making contraction furrows poorly visible; (**D**) iris surface reconstruction using SS-OCT with contraction furrows that are much more clearly visible.

**Figure 2:**
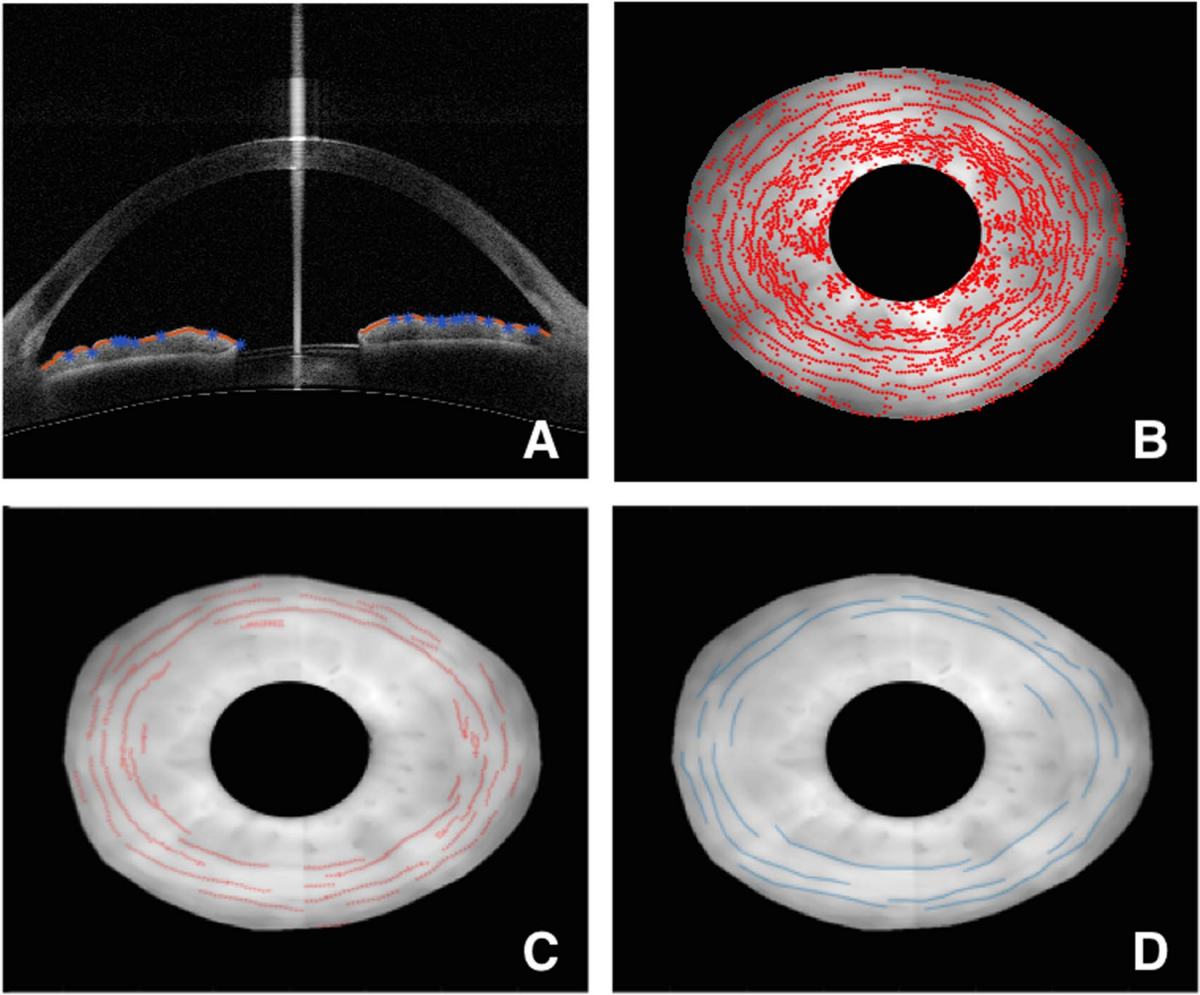
Measurement of furrow length using SS-OCT; (**A**) Extrema (minima) points (blue) on the anterior iris boundary (red) are identified as candidate furrow points; (**B**) Candidate furrow points overlaid on the reconstructed iris surface reveal clusters of discontinuous points near the pupillary boundary and continuous lines corresponding to furrows in the periphery; (**C**) Final furrow points after filtering based on thresholding to reject clustered and discontinuous points; (**D**) Manual markings of furrows on the same eye.

There are several features that can be found in the surface of the human iris, including crypts and contraction furrows. Crypts are regions of iris stroma hypoplasia or atrophy that stereoscopically appear as depressions on the iris surface (Fig. 1) whereas contraction furrows are regions on the iris that are circumferentially folded when the iris adapts to different light conditions (Fig. 2). Our group developed an iris surface grading system for Asian eyes^16^, and recently showed that iris surface features, such as crypts, furrows and color are associated with iris morphology, such as its thickness^16^ and volume^17^. Specifically, the presence of contraction furrows was significantly correlated with a thicker iris^16^. We hypothesized that iris surface features may be surrogates for iris stromal characteristics, affecting the dynamic changes in iris volume. Based on our iris grading system, we recently identified that a lower iris crypt grade (fewer crypts) was associated with a smaller iris volume change during physiological pupil dilation. However, we failed to observe a significant association between iris furrow grades and dynamic changes of iris volume. A possible reason for this is that the assessment of iris furrows was based on a three-point grading scale^16^. While this grading system has allowed us to identify significant associations with static iris thickness^16^ and volume^17^, having only 3 grades may be less sensitive in capturing the full range of variations in iris characteristics among study subjects.

In the present study, we developed an automated tool to measure iris furrow length using images acquired from anterior segment swept-source optical coherence tomography (SS-OCT). Our novel method was able to provide an objective and more quantitative measurement of the actual length of iris furrows, and thus is more sensitive to capture the full variations in furrow length. Furthermore, we applied this new technique on a set of study eyes and were able to identify an association between iris furrow length and changes in iris volume during physiological pupil dilation.

## Methods

### Participants

Subjects of this study were enrolled from a larger community-based study conducted between June and September 2013, involving more than 2000 volunteers aged 50 years older, who underwent a standardized eye examination at a community polyclinic^14^. At the time of enrolment, individuals visited the polyclinic for minor health issues (non-ocular) and did not have any ophthalmic complaints during consultation. A total of 300 of these participants with no history of ocular disease and normal ocular findings on the basis of eye examination (see details below) were subsequently referred back to Singapore Eye Research Institute (SERI) for a follow-up eye examination, conducted between June 2013 and August 2014. For this study, we prospectively recruited 86 consecutive participants from the participants who attended the follow-up examination between June and August 2014 for SS-OCT imaging (see later section).

Each participant underwent an interview and a detailed ocular examination based on a standardized study protocol, including visual acuity measurement using Logarithm of Minimum Angle of Resolution (LogMAR chart, The Lighthouse, NY), automated refraction to assess refractive error, intraocular pressure (IOP) measurement using non-contact tonometry, iris photography (see later section), slit-lamp examination of the anterior segment (Haag-Streit, Bern, Switzerland), gonioscopy, and optic disc examination through an undilated pupil by an ophthalmologist.

Subjects were considered to have normal findings if they had presenting LogMAR visual acuity (VA) of 0.3 (20/40) or better in either of the eyes, absence of ocular conditions such as glaucoma, cataract (any Lens Opacities Classification System II grading >2), retinal or ocular comorbid conditions including, but not limited to, diabetic retinopathy and age-related macular degeneration. The study was approved by the SingHealth Centralised Institutional Review Board and conformed to the tenets of the Declaration of Helsinki. Written Informed consent was obtained from all participants.

### Iris Photography and Iris Crypt Grading

Color photographs of both eyes’ irides were taken using an iris imaging system (MEC-5-ASL-D7100-N85, Miles Research, CA, USA) that consisted of a 24 megapixel Nikon Camera (Nikon D7100, Nikon, Japan), Nikon 85 mm macro lens (Nikon D3200, Nikon, Japan), adjustable side lighting illuminator (MEC-5-ASL, Miles Research, CA, USA), and chinrest/camera support (CRCS-FH4, Miles Research, CA, USA). Photographs were taken in a room where room fluorescent lighting was kept on. Biometric coaxial illuminators were angled 60° and used to deliver light to the iris at a constant illumination in order to maintain color and brightness. The camera setting was kept constant at: aperture priority dial, aperture stop (*f*18) shutter speed (1/60”), ISO (200), flash power (1/2), focal length (1 ft/0.286 m). For grading purposes, photographs were viewed on a 1366 × 768/60 Hz resolution screen, using the viewing software ACDSee Photo Manager Version 11.0 (ACD Systems, Washington, USA).

Iris crypts were graded as described previously^16^. In brief, irides were given an integer grade between 1 to 5 based on the number and size of crypts present as follows: Grade 1 (no crypts); Grade 2 (1 to 3 crypts); Grade 3 (at least 4 crypts ≤1 mm in diameter); Grade 4 (at least 4 crypts >1 mm in diameter); and Grade 5 (numerous crypts >1 mm in diameter, covering nearly the entire iris).

### Swept-Source Optical Coherence Tomography Imaging

The study participants underwent imaging using SS-OCT (Casia SS-1000 OCT; Tomey, Nagoya, Japan), before any contact procedure or eye drops, under standardized illumination conditions as detailed below. To avoid eyelid artifacts, the operator opened both eyelids, avoiding inadvertent pressure on the globe during scanning. Participants were directed towards an internal fixation target and each eye was scanned with the 3-dimensional angle analysis scan using the auto alignment function. Each SS-OCT scan took 2.4 seconds to complete and was comprised of 128 radial cross-sections that were 16 mm in length and 6 mm in depth. The resolution of each cross-section was 9.7 μm in depth. All eyes were first imaged in the dark followed by the light condition. Under dark conditions, room lights were turned off and no stray light was allowed in either eye. The light condition was comprised of both room illumination and a bright flashlight directed at the second, non-imaging eye during the scan. Both eyes of each participant were imaged in the dark followed by light condition with a duration of 2 minutes between scans.

### Measurement of Iris Volume

Iris volume and iris furrow length were measured using a custom-written program on MATLAB (Mathworks Inc., Natick, MA, USA). The program automatically detected iris boundaries on all 128 cross-sections of each SS-OCT scan and replaced the need for manual input, making the measurement process quick and efficient.

The methodology of iris volume measurement was identical to that described in our previous paper^18^. Briefly, for each of the 128 cross-sections making up one SS-OCT scan, the anterior and posterior boundaries of the iris were detected using a gradient-based thresholding algorithm. Once the iris boundaries were identified on all 128 cross-sections, a 3-D reconstruction of the entire iris was generated to allow for measurement of iris volume as well as visualization of the iris surface (Fig. 1A and B). Based on this reconstruction, iris volume was defined as the volume enclosed by the space between the anterior and posterior iris surfaces. Mathematically, this amounted to counting all the voxels enclosed within this space. The dimensions of each voxel are shown in Fig. 1B. Based on these dimensions, the volume of each voxel is θr pixel^3^. In this study, $\theta =\pi /128$ (i.e., analysis was performed every 1.4°). Substituting for the value of θ and summing up the volumes of all contributing voxels, we get iris volume to be: $\pi /128{\sum }_{s=1}^{128}{\sum }_{i=1}^{n}{r}_{si}{(scalingfactor)}^{3}$, where *s* represents the scan number, *n* represents the pixel number of the iris in a B-scan (or brightness scan; representing one cross-sectional imaging scan of the 128 cross-sections scans), and *r*_*si*_ represents the distance of the pixel from the center. We numbered all pixels contained between the anterior and posterior surfaces in a single cross section. The voxel number incorporates both co-ordinates in a 2D B-scan (see Supplementary Fig. S1). Hence, index ‘i’ will count the entire pixel that belongs to the iris in a B-scan. The scaling factor was 9.7 μm/pixel. In a minority of images (1.6%), manual adjustments were made if the software failed to automatically detect the iris and corneal boundaries at the correct location. Pupil diameter (PD) was also measured using our program, defined as the mean distance between the two pupil edges across all 128 cross-sectional scans.

### Measurement of Iris Furrow Length

Using SS-OCT to measure furrows makes them easier to detect due to better visibility when compared to digital iris photographs (Fig. 1C and D) and also deepens our understanding of how furrows manifest themselves in 2-D photographs by allowing for 3-D visualization.

Furrows were identified using the delineated anterior iris boundary. Along individual B-scans, furrows manifest themselves as short valley-like depressions on the anterior surface. Candidate furrow points were first identified as locally deep (minima) points on the detected anterior iris boundary along each of the 128 B-scans (Fig. 2A). Next, candidate furrow points were overlaid on the reconstructed iris surface and filtered based on the likelihood of their being either a furrow or just random noise (Fig. 2B). Furrows are typically circumferential and present along the periphery. This biological property was exploited by applying a threshold to filter out candidate furrow points: (1) points farther from the center of the pupil were more likely to be from a furrow and were given a greater weight; (2) points that were circumferentially continuous were more likely to be furrows and so isolated, discontinuous points were rejected. The filtering was carried out in the following way. After candidate furrow points from the B-scans were overlaid on the reconstructed enface iris surface, points were stretched circumferentially, with those further away from the pupil center being stretched to a greater degree. A region around detected furrow point is defined and any other point lies within this region belongs to the same furrow. Points are stretched circumferentially and radially, meaning that radius was allowed to vary within a furrow segment. Circumferentially, stretching is 1.2 times the angular distance between successive B-scans. Radially, the stretching distance is 45 pixels on either side of the point, which corresponds to a band that is about 0.9 mm wide. An illustration of the region surrounding each point is shown in Supplementary Figure S2. The extent of stretching was proportional to the distance of the point from the center of the pupil and reflected the fact that B-scan points further away from the center contribute a greater circumferential distance in the 3-D reconstructed iris (Fig. 1B). Stretching also allowed neighbouring points to overlap, creating continuous lines. Next, the lengths of these continuous lines were measured, and those lines shorter than 0.3 mm were considered noise and excluded. This 0.3 mm threshold was determined based on comparisons with manually delineated furrows on a sample of 10 SS-OCT scans (Fig. 2C and D). The length threshold that gave optimal agreement between automated and manual furrow measurements was chosen. For each eye under light and dark conditions, the lengths of detected furrows were added up and total furrow length was reported in millimetres.

### Agreement of Iris Furrow Length Measurements

Accuracy of the furrow length detection was assessed by measuring the percentage overlap between two detections made on the same SS-OCT scans. Two observers (ST and RL) independently marked furrows on a set of 20 eyes using a custom-written manual delineation tool on MATLAB. These manual delineations were compared to the automated detections and the percentage of furrow lines that overlapped between the two was reported (using manual markings by ST as the baseline) (Fig. 2C and D). In addition to the agreement between manual and automated detections, intra- and inter-observer agreements were also reported.

### Statistical Analysis

Linear regression analysis was used to assess the associations between iris furrow length and both static and dynamic iris volume. Potential confounders, such as age, gender, pupil size and presence of crypts were included in the multiple linear regression models. Pupil size within the light or dark conditions varied between eyes and it has been shown to be associated with iris thickness. The presence of crypts has also been shown to be associated with iris thickness. Crypt measurement was based on the grading scale used in our previous studies^7,16,18^. There was a strong inter-eye correlation in the measurements of iris volume change (r = 0.90, P < 0.001). Data from right eyes were used in our analysis to eliminate the inter-eye correlation issue. Analysis was performed using STATA 12.1 (StataCorp LP, College Station, Texas). For assessing the association with change in iris volume after pupil dilation, we also used the change in iris volume per pupil size change (in mm) as an outcome measure^13^.

## Results

Of the 86 Chinese participants originally enrolled, 5 were excluded due to poor SS-OCT scans (eye movements resulting in missing or poorly registered B-scans). In addition, 5 were excluded due to inability to grade iris crypts due to corneal arcus, and 11 had narrow angles (i.e. AOD 750 ≤ 225 μm). The remaining 65 participants with open angles (75.6%) were included in the final analysis. The mean age of the included participants was 59.8 ± 5.7 years, and 42 (64.6%) were female (Table 1).

**Table 1:** Demographics and baseline characteristics of Chinese participants included in the study (n = 65).

Characteristics	Mean (Standard Deviation) or No. (%)
Age, years	59.84 (5.67)
Gender	
Male	23 (35.38)
Female	42 (64.62)
Intraocular pressure, mmHg	14.34 (2.76)
Vertical cup-to-disc ratio	0.37 (0.10)
Angle opening distance, mm	0.41 (0.13)
Pupil diameter, mm	
Light	2.79 (0.60)
Dark	3.92 (0.79)
Change (dark *minus* light)	1.13 (0.48)
Iris volume, mm^3^	
Light	38.00 (3.56)
Dark	36.81 (3.79)
Change (dark *minus* light)	−1.19 (0.66)
Furrow length, mm	
Light	43.44 (27.34)
Dark	57.63 (24.61)
Change (dark *minus* light)	15.84 (13.59)

In validating our furrow length measurements against the baseline manual markings (ST), the automated detection overlapped with 76% of the furrows. The overlap between the second observer’s (RL) markings and the first observer’s baseline markings was 82% (0.82). Regarding the intra-observer agreement, the overlap was considerably higher at 95%. We assessed the reproducibility of the measurements with repeated imaging on a set of 10 eyes. We obtained a Pearson’s correlation coefficient, r = 0.93, P < 0.001 and an intraclass correlation coefficient of 0.88 (95% CI, 0.62–0.97) between the first and second measurements, suggesting excellent agreement.

Going from light to dark condition, the study eyes’ pupils dilated by an average of 1.13 mm (range, 0.32–2.42 mm). The mean iris volume in the light condition was 38.00 mm^3^ and eyes showed a reduction (−1.19 ± 0.66 mm^3^, P < 0.001) in iris volume going from the light to dark condition. Iris furrow length in the light condition was 43.44 ± 27.34 mm and increased significantly when going to the dark condition (15.84 ± 13.59 mm, P < 0.001) (Table 1).

Longer furrow length in the light condition was marginally significantly associated with a larger iris volume in the light condition (β** = **0.37; P = 0.05 adjusted for age, gender and pupil size) and the association was more significant with a larger iris volume in the dark condition (β** = **0.53; P = 0.007 adjusted for age, gender and pupil size; Model 1 of Table 2). Since the iris crypts were also shown to be associated with iris volume^18^ we included a second model where we additionally adjusted for the crypt grade, and the results were largely similar (Model 2 of Table 2).

**Table 2:** Associations between furrow length and static iris volume.

	Model 1*		Model 2^†^	
	β (95% CI)	P value	β (95% CI)	P value
Iris volume in light condition	0.37 (0.00, 0.73)	0.050	0.33 (−0.00, 0.67)	0.054
Iris volume in dark condition	0.53 (0.15, 0.91)	**0.007**	0.41 (0.05, 0.77)	**0.028**

β, change in iris volume (in millimeters^3^) per 10 mm increase in iris furrow length; CI, confidence interval.

*Model 1: β was adjusted for age and gender for both outcome measures, and additionally adjusted for 1) pupil size in light for assessing iris volume in light condition, and 2) pupil size in dark for assessing iris volume in dark condition. Pupil size was measured from SS-OCT images.

^†^Model 2: In addition to the covariates included in Model 1, β was additionally adjusted for iris crypt grade.

With regard to dynamic changes of iris volume, longer furrow length in the light condition was significantly associated with a smaller reduction in iris volume after pupil dilation (β** = **−0.10, representing 0.1 mm^3^ smaller reduction in iris volume per 10 mm increase in iris furrow length, *P* = 0.002 adjusted for age, gender and change in pupil size). Using change in iris volume per pupil size change (in mm) as the outcome, we obtained consistent findings: a 10 mm increase in furrow length was associated with a 0.09 mm^3^ smaller reduction in iris volume per pupil size change in mm (P = 0.002 adjusted for age and gender). The associations also remained similar in the second model where we additionally adjusted for crypt size (Table 3).

**Table 3:** Associations between furrow length and dynamic iris volume change.

	Model 1*		Model 2^†^	
	β (95% CI)	P value	β (95% CI)	P value
Change in iris volume after pupil dilation	−0.10 (−0.15, −0.04)	**0.002**	−0.09 (−0.14, −0.03)	**0.005**
Change in iris volume per pupil size change (in mm) after pupil dilation	−0.09 (−0.14, −0.03)	**0.002**	−0.08 (−0.14, −0.03)	**0.004**

β, change of the outcome measures (in millimeters^3^) per 10 mm increase in iris furrow length; CI, confidence interval.

*Model 1: β was adjusted for age and gender for both outcome measures, and additionally adjusted for change in pupil size between light and dark conditions for assessing change in iris volume. Pupil size was measured from SS-OCT images.

^†^Model 2: In addition to the covariates included in Model 1, β was additionally adjusted for iris crypt grade.

## Discussion

In this study, we introduced a novel technique to detect iris surface furrows using SS-OCT scans of the anterior segment of the eye. Our methodology allows for furrows to be detected objectively and automatically and demonstrated good agreement with manual delineations. In addition, we utilized this automated furrow length measurement technique from OCT images to answer a clinically relevant question: Can iris furrows predict the change in iris volume, a risk factor for angle closure disease? We applied this technique on a sample of normal Chinese subjects with open-angles and found that eyes with longer furrows tend to exhibit a smaller change in iris volume during physiological dilation.

Iris furrows have traditionally been assessed using a manual grading system from digital iris photographs^7,16,19,20^. Such a method is limited by the presence of arcus senilis, and offers only three grades. Our newly proposed method detects and measures furrows on a continuous furrow length variable in millimetres, and is therefore more informative. Interestingly, iris furrows were more visible in our reconstruction of the anterior segment SS-OCT scans than the iris photographs (Fig. 1C and D). Moreover, we have demonstrated that our new technique shows good agreement with manual delineations.

**Figure 3:**
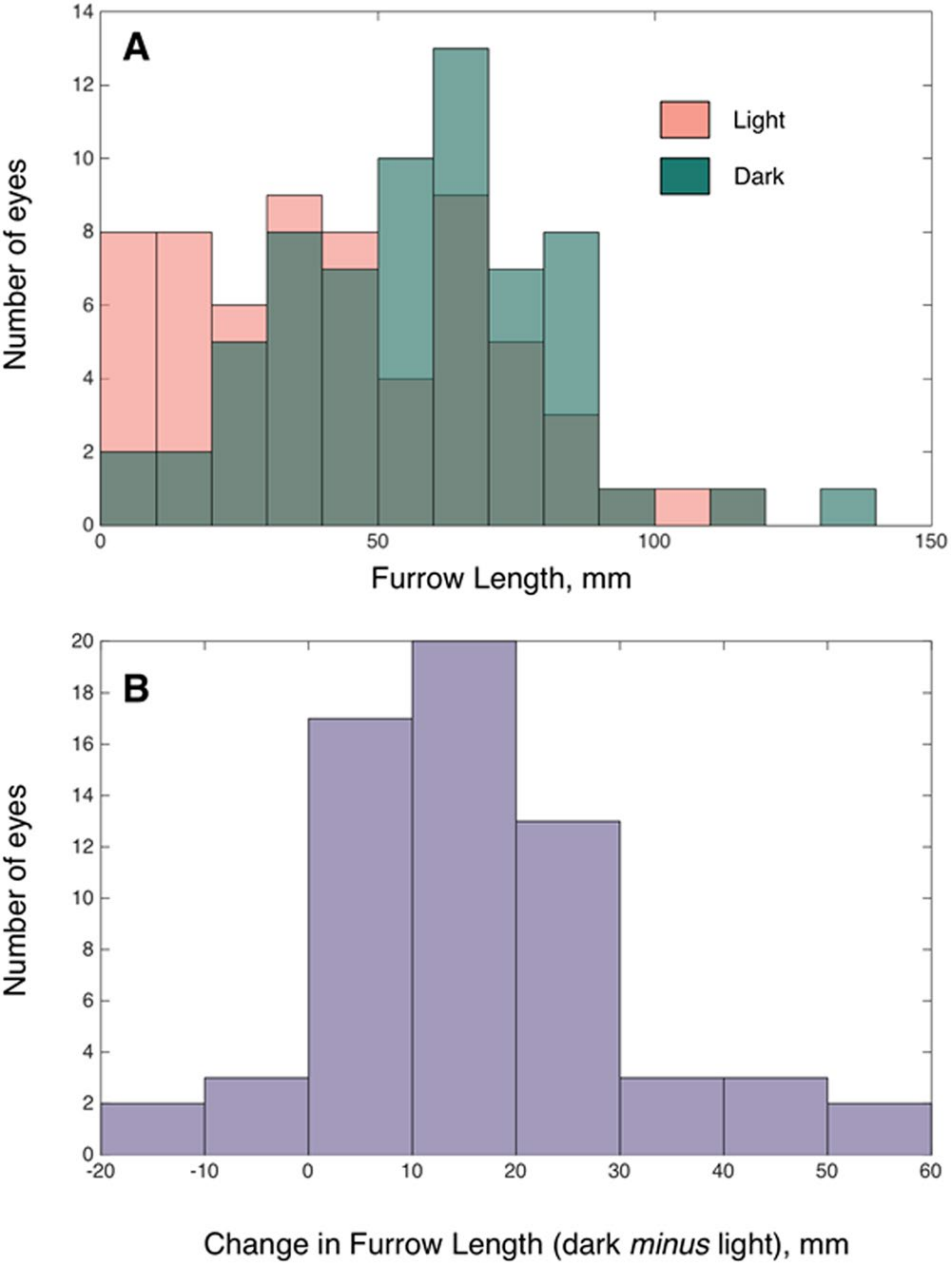
(**A**) Distribution of iris furrow length in light (pink) and dark (green) conditions; (**B**) Distribution of change in furrow length; furrow length increases with pupil dilation (light to dark) for a majority of the eyes.

Contraction furrows are regions on the iris that are circumferentially folded when the iris adapts to different light conditions. It is expected that with pupil dilation, the iris will contract and hence fold up to a greater degree. Using our measurement of furrow length, we are for the first time able to show that this is indeed the case: going from the light to dark condition, wherein the pupil dilates, there is a significant increase in furrow length (Fig. 3).

Based on the morphological origins of furrow formation, it has been suggested that the density of the iris, including all five of its layers, could influence the extension and distinction of furrows^19,21^. Thus, furrows could be seen as a surrogate for iris stromal characteristics such as density of connective tissue, presence of water, and bonding forces. Moreover, it is believed that the iris acts like a sponge, absorbing aqueous humor from the posterior segment of the eye and releasing it into the anterior chamber, thereby enabling its free flow^22–25^. Reduction in this fluid exchange across the iris could create a risk for angle closure disease. We speculate that an iris with more and/or longer furrows is less sponge-like (denser, lower water content) and might lead to reduced fluid exchange during pupil dilation. This may explain our finding that eyes with longer furrow length tend to have a smaller reduction in iris volume after physiological pupil dilation. Previous studies on dynamic iris volume changes have reported that fellow eyes of eyes with prior acute primary angle closure (APAC) exhibit smaller change in iris area during pupil dilation when compared to primary angle closure suspects (PACS)^10,15,26^. This suggests that furrow length could also be linked to angle closure disease. However, further studies on eyes with narrow angles would be needed as our present study only investigated the association of furrow length and change in iris volume in normal eyes with open angles.

Our findings offer greater evidence for the hypothesis that iris surface features reflect underlying iris structural characteristics and influence the dynamic changes in iris morphology during pupil dilation. We recently also showed that the presence of more crypts was associated with smaller iris volume and greater change in iris volume during physiological pupil dilation^18^. The associations with furrow length remain significant even after adjusting for the presence of crypts, suggesting that the two iris surface features – crypts and furrows – may have an independent influence on dynamic changes in iris volume.

There are some limitations in this study. First, all participants included in this study had normal open drainage angles. Further investigation is needed to study the association between furrows and dynamic iris volume change in subjects with angle closure disease. Second, our methodology involved physiological pupil dilation. While this reflects the natural range of pupil activity, pharmacologic mydriasis can extend the range of pupil dilation, thereby allowing for additional analysis. Third, our measurement of furrows required high-resolution (128 cross-sections) 360° SS-OCT scans to be performed on the eye. While each scan is relatively quick (2 seconds), it was still long enough for there to be registration issues, resulting in misalignment of the first and last radial B-scans in the 3D reconstruction of the iris (seen nasal-temporal direction in Fig. 1D). This resulted in discontinuities in furrow detection across the misaligned B-scans (Fig. 2C). This issue was however only among eyes with significant movement during the scan. Future advances in SS-OCT may allow for faster scans, reducing scan durations and subsequent registration issues. Finally, SS-OCT, while becoming increasingly more accepted, still remains limited in use and may not be accessible in every clinical setting. Thus, while our methodology offers a robust way of measuring furrows in a research setting, more work needs to be conducted in order to allow furrow detection to be more feasible (such as a quantitative measurement based on iris photographs) in a clinical setting.

In conclusion, we have introduced a novel way of measuring iris furrows *in-vivo* using SS-OCT. Our measurements are robust, reliable and sensitive to subtle changes, such as during pupil dilation. In our study sample, we found that the presence and length of iris furrows affect the changes in iris volume during physiological pupil dilation. Our findings suggest that contraction furrows on the surface of the iris may be linked to underlying iris morphology.

## Electronic supplementary material

Supplementary Figures
