# Supplementary material for: Automated Detection of Iris Furrows and their Influence on Dynamic Iris Volume Change
Source: Sci Rep. 2017 Dec 20;7:17894. doi: 10.1038/s41598-017-18039-w (PMC5738384; doi:10.1038/s41598-017-18039-w)
Supplement: Supplementary file 1 — Supplementary Figures [file 41598_2017_18039_MOESM1_ESM.pdf]

Automated Detection of Iris Furrows and their Influence on Dynamic Iris Volume Change

Jacqueline Chua, BOptom, PhD,[1,2] Sri Gowtham Thakku, BEng,[1] Tan Hung Pham, BEng,[1,3] Ryan Lee,[1,4] Tin A Tun, MD,[1] Monisha E. Nongpiur, MD, PhD,[1,2] Marcus Chiang Lee Tan, MBBS,[1] Tien Yin Wong, FRCS, PhD,[1,2,4] Joanne Hui Min Quah, MMed,[5] Tin Aung, FRCS, PhD,[1,2,4] Michael J. A. Girard, PhD[1,3,+] Ching-Yu Cheng, MD, PhD[1,2,4,+]

[1] Singapore Eye Research Institute, Singapore National Eye Centre, Singapore

[2] Ophthalmology & Visual Sciences Academic Clinical Program (Eye ACP), Duke-NUS Medical School, Singapore

[3] Department of Biomedical Engineering, National University of Singapore, Singapore

[4] Department of Ophthalmology, Yong Loo Lin School of Medicine, National University of Singapore and National University Health System, Singapore

[5] SingHealth Polyclinics, Outram, Singapore

[+]Contributed equally

Corresponding author:

Dr Ching-Yu Cheng

20 College Road, The Academia, Level 6, Discovery Tower Singapore 169856

Tel: +65 65767277, Fax: +65 6225 2568

Email: chingyu.cheng@duke-nus.edu.sg

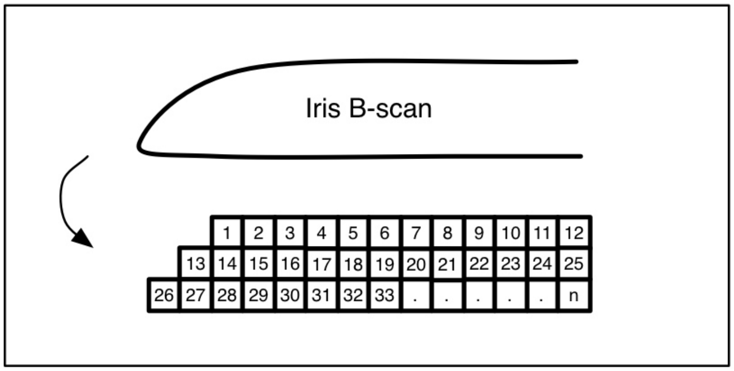

Supplementary Figure S1: Voxel number 'i' ranging from 1 to n for a given B-scan image.

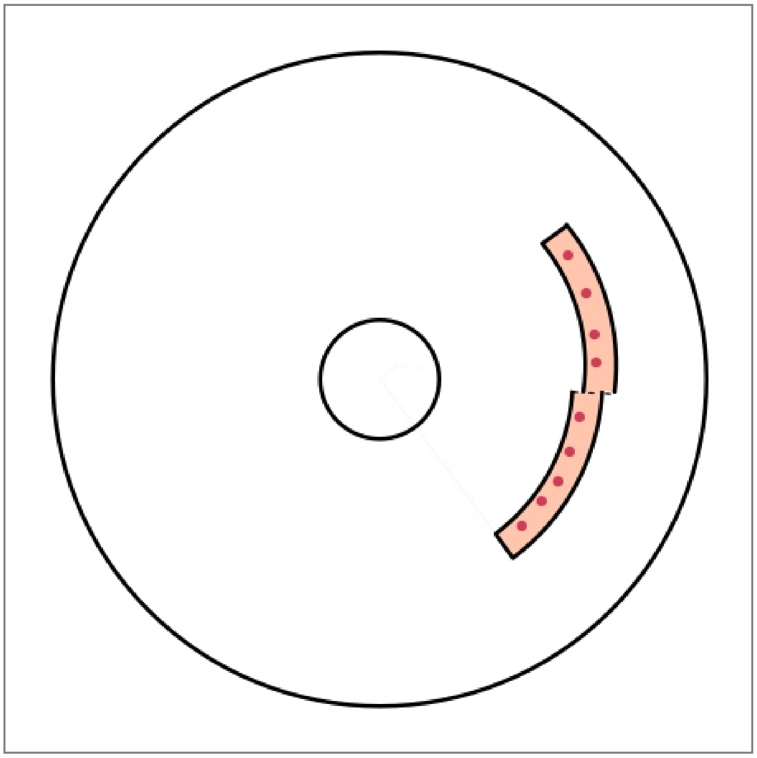

Supplementary Figure S2: Furrow matching tolerance; red dots correspond to identified furrow points; the pink region around the dots defines the acceptable matching tolerance for furrows - if pink regions overlap, the points are defined as belonging to the same furrow.
